# A National Survey of postgraduate physician assistant fellowship and residency programs

**DOI:** 10.1186/s12909-021-02613-y

**Published:** 2021-04-14

**Authors:** Vasco Deon Kidd, Sarah Vanderlinden, Roderick S. Hooker

**Affiliations:** 1 Director of Advanced Practice Providers, The University of California, Irvine, UCI Health, Orange USA; 2 Assistant Director Advanced Practice, APP Critical Care Fellowship Program, Medical College of Wisconsin, Department of MCP Clinical Affairs, Milwaukee, USA; 3 Adjunct Professor of Health Policy, Northern Arizona University, Flagstaff, USA

**Keywords:** Physician associate, Advance practice registered nurse, Academic medical centers, Residency, Fellowship, House officers

## Abstract

**Introduction:**

The development of postgraduate programs for physician assistants (PAs) began in 1973 and by 2020 there were approximately 72 programs spread across a broad range of medical and surgical disciplines. PA Post-graduate education programs are voluntary and available to American licensed PAs. Therefore, an assessment of the characteristics of PA post-graduate fellowships and residencies programs was initiated.

**Method:**

A non-experimental, descriptive research study was designed to obtain information on the characteristics of PA postgraduate education programs in the US. The source of information was from surveyed members of the Association of Postgraduate Physician Assistant Programs (APPAP). Questions were drawn from consensus discussions. Directors of postgraduate programs that were operational in 2020 were eligible to participate.

**Results:**

Seventy-two postgraduate program directors were invited to the survey and 34 program directors replied. These programs are geographically distributed across the US in 13 states. The respondents represent a wide range of medicine: surgery, emergency medicine, critical care, orthopaedics, hospitalist, psychiatry, oncology, primary care, pediatrics, and cardiology. Most programs are associated with an academic medical center and some institutions have more than one postgraduate specialty track. The curriculum includes bedside teaching, lectures, mentorship, assigned reading, procedures, simulation, and conferences. An average program length is 12 months and awards a certificate. Stipends for PA fellows are $50,000–80,000 (2020 dollars) and benefits include paid time off, health and liability insurance. About half of the programs bill for the services rendered by the PA. Over 90% of graduates are employed within 2 months of completing a PA postgraduate training program.

**Conclusion:**

A trend is underway in American medicine to include PAs in postgraduate education. PA postgraduate training occurs across a broad spectrum of medical and surgical areas, as well as diverse institutions and organizations overseeing these programs. Most PA postgraduate programs are in teaching hospitals where the PA resident or PA fellow also serves as a house officer alongside a categorical resident. This study sets the stage for more granular economic and social research on this growing phenomenon in American medicine.

**Supplementary Information:**

The online version contains supplementary material available at 10.1186/s12909-021-02613-y.

## Introduction

Postgraduate education for American physician assistants (PAs) has been underway for half a century. Montefiore Hospital in New York introduced PAs as house officers on the surgery service in 1971 and began the first PA postgraduate fellowship program in 1973. Norwalk Hospital/Yale School of Medicine in 1976 was next [1, 2]. In the second decade Mount Sinai Hospital PA Surgical Program, University of Oklahoma Occupational Medicine PA Residency, and The University of Southern California Medical Center Emergency Medicine PA Residency followed. Other postgraduate programs were created for intensive clinical experience in rural medicine, neonatology, and primary care [3]. By the new century postgraduate programs had broadened into many areas of medical and surgical specialties.

For 50 years a trend has been underway to adopt a postgraduate model of PA education [4]. The Association of Postgraduate PA Programs (APPAP.org) website lists 72 programs spanning 32 tracks - some within the same institution [5]. Additionally, some programs were paused during the COVID-19 pandemic of 2020–21.

PA education was established in the 1960s, due to limited access to healthcare services and physician supply. Spanning half a century the PA movement in 2020 numbered over 140,000 clinically active PAs and the 265 programs produce almost 10,000 graduates [6, 7]. This growth is only matched by nurse practitioners (NPs). Less than 1 % of PA graduates in any year attend a postgraduate program. Nevertheless, the growth in the number of postgraduate programs appears to be rising.

To set the stage for this project the medical school postgraduate pipeline has largely been constrained due to a federal policy in 1997 that restricted graduate medical education growth. National restriction on resident and fellow work hours per week has also limited the supply of house officers at a time of hospital expansion and consolidation [8]. The medical physician house officer shortage occurred at the same time PA visibility became more prominent. In addition, expanding healthcare teams and coverage models to include NPs and PAs has been shown to increase access to quality patient care services at lower total costs [9]. Formalized postgraduate programs have provided one pathway for transition-to-practice through hands-on clinical experience and structured education, especially in departments where it may be challenging to onboard new graduates to provide highly specialized care. Additionally, PA post-graduate training programs may provide value to the sponsoring institution in terms of diversified recruitment, retention of staff, interprofessional collaboration, and professional development opportunities, including teaching and assessment of postgraduate PA trainees [5, 10].

Postgraduate PA programs have been insufficiently researched and more clarity is needed about this aspect of medical education [5]. The notion that PAs can supplement house officers (i.e., physician residents or fellows) is growing as shown by the number of new and established programs. However, little information has been advanced in terms of the broad characteristics of PA postgraduate programs. Informing administrators, researchers, and medical directors about this trend is warranted. Documenting when, where, why, and how PAs and nurse practitioners (NPs) are used in American medicine is important to health economists and workforce planners if national goals of access to care are to be met [11]. The objective of this study is to identify the current state of PA postgraduate programs in America**.** To address this, a survey of PA postgraduate education programs was undertaken.

## Method

A non-experimental, descriptive research study was designed to obtain aggregated information on the characteristics of PA postgraduate education programs in the U.S. The list of programs was drawn from the APPAP membership (APPAP.org). The APPAP website contains information on each program, the sponsoring institution, a contact, degree awarded, and class size. Additional information is gained through reaching the program’s website.

Questions were developed based on the collective experience of the authors with postgraduate programs and inquiries to APPAP board members. Two pilot surveys were distributed to 4 postgraduate program directors for review and comment. Feedback and suggestions improved the instrument. The 2019 survey results were discussed at the APPAP open annual business meeting with board members and program directors who supported a refined 2020 survey. Through this open, iterative process, the questions were refined, and the 2020 survey finalized. Fifty-nine questions were selected. The survey was organized into 7 main sections. The survey instrument was uploaded in Survey Monkey©, an on-line social science survey firm. The web-based questionnaire invitation was sent to PA postgraduate program directors who are active members of APPAP during October 2020 and two separate reminders followed. Each question was singular and not algorithm driven. Responses were aggregated and descriptive statistical analyses were utilized through a statistical package built into the survey software. Text space below the questions provided for impromptu comment by the respondent. The estimated time to complete the questionnaire was 17 min. The survey was an APPAP Board of Directors (BoD) initiative and considered an administrative project undertaking for continuous quality and process improvement. This project did not involve special populations. Additionally, this information can be found in the public domain and therefore the need for an institutional review board was not explored.

## Results

In total 72 PA postgraduate programs directors were sent a set of questions along with an introduction for participation. Thirty-four program directors (47% rate of return), responded to the survey questions. On several occasions, the respondent opted to skip a question and the (per question response rates varied between 20 and 100%). Additionally, the survey also included several open-ended questions, which provided respondents the opportunity to give additional feedback. The 34 postgraduate programs are spread across the US in 13 states. Most programs are associated with an academic medical center. Because some directors oversee more than one specialty track within an institution, the total number of postgraduate programs represented in the answers varied.

**Fig. 1:**
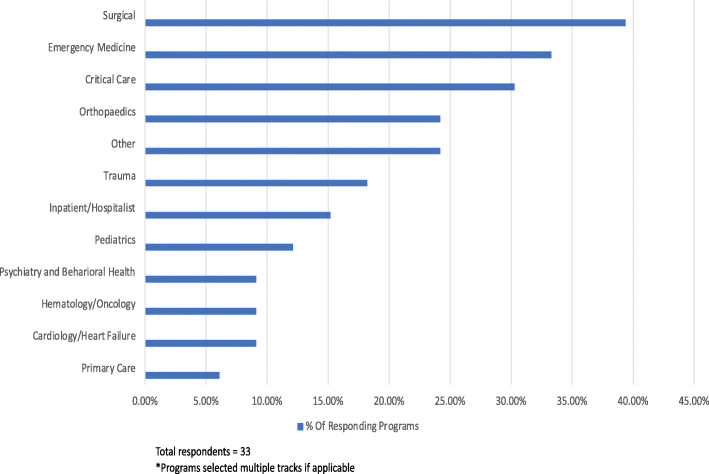
PA Postgraduate Program Specialty Tracks

The majority (82%) of the 34 respondents represent a single-track program, meaning they provide postgraduate training in one discipline such as orthopaedics; 18% are multi-track programs. The disciplines most represented are surgical (39%), Emergency Medicine (33%), Critical Care (30%), and Orthopaedics (24%). (Fig. 1).

All represented postgraduate programs are focused on the education of PAs, and 12 (35%) include nurse practitioners (NPs). The duration of most postgraduate PA education is 12 months (79%), with two at 18 months, and one two-year program. All award a certificate of completion. Some programs include research and award a postgraduate degree [12]. A majority (89%) of 33 responses reported PA fellows received education alongside physician residents in the same specialty.

**Fig. 2:**
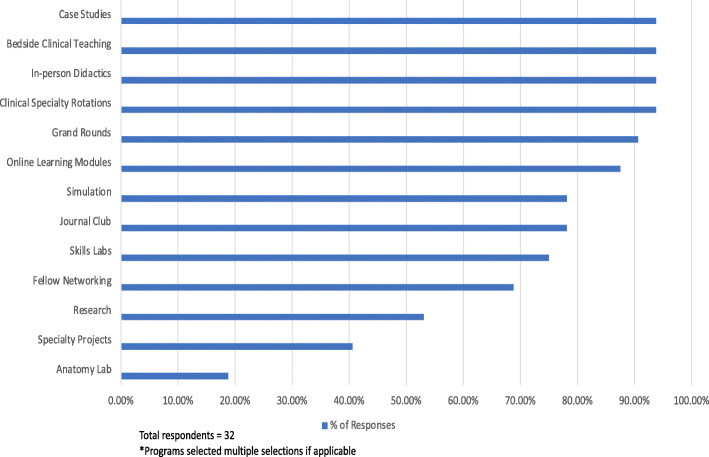
PA Postgraduate Program Education Delivery Methods

The techniques used by postgraduate programs in PA role development varies. The most common education delivery methods are case studies, bedside clinical teaching, in-person didactics, specialty rotations, grand rounds, online learning modules, simulation, and journal clubs (Fig. 2**).**

**Fig. 3:**
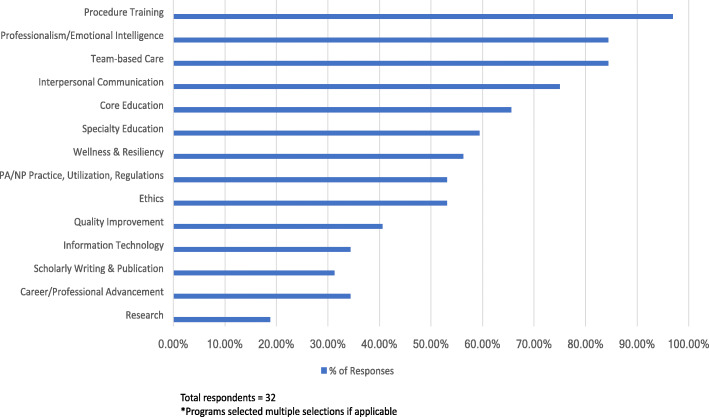
PA Postgraduate Program Curriculum

Beyond a focus on the education about the particular medical or surgical specialty involved, the program incorporates a wide variety of skill development activities such as procedure training, professionalism and emotional intelligence, roles in team-based care, and interpersonal communication. **(**Fig. 3**)** Nearly all programs provide continuing medical education credits for maintenance of national certification and state licensure. Programs identified using electronic systems to deliver education (91%). All PAs must be licensed in the state where employed.

**Fig. 4:**
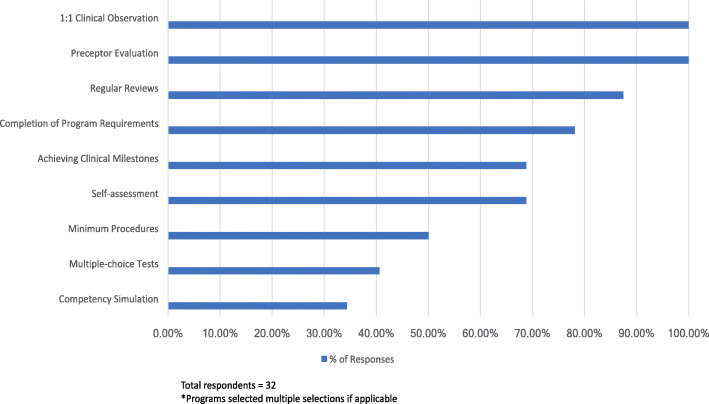
Methods for Assessing PA Fellow Performance

Evaluations and assessments of PA fellows incorporate various methods. The more common techniques are one-on-one observations by a mentor or preceptor, instructor evaluations, through regular reviews with the program director or supervising (e.g., attending) physician, completion of program requirements, and achieving clinical milestones (Fig. 4).

### Institutional affiliation

Administratively half (55%) of PA postgraduate programs reside in medical schools, with (40%) in health science colleges, and one (5%) in a nursing school. Three-quarters (76%) of the hospitals that sponsor PA postgraduate programs are affiliated with an academic medical center. The others are vertically integrated multi-hospital systems such as The Mayo Clinic (located in three states) (15%), community hospitals (6%), and one in an ambulatory clinic. Nearly all PA postgraduate programs (94%) credentialed and privileged the PA fellows similarly to other PAs and NPs in the organization. Almost all PA postgraduate programs (97%) award a certification of completion or a diploma (3%). The combined Army/Air Force – Baylor University program awards a Doctor of Science (DSc) for active-duty members and Arrowhead Orthopedics, in collaboration with University of Lynchburg, offers a Doctor of Medical Science (DMSc) track [12].

### Nomenclature

PAs in postgraduate training are referred to as “Fellows” (59%) or “Residents” (41%). One respondent commented, “We refer to them as *fellows*, but our program is HRSA grant-funded which is NP only and requires the name *resident*.”

### Applicant pool

Most programs (88%) draw on a national applicant pool with the remainder relying on regional or local applicants. All PAs accepted into a program are graduates of an accredited program and all are nationally certified by the National Commission on the Certification of Physician Assistants (NCCPA). Entry into a postgraduate PA program most commonly occurs within 2 years of graduation.

### Employment of PA postgraduate graduates

Upon graduation from a postgraduate training program, the respondents reported that (96%) were employed within 2 months. When asked about the employment opportunity for graduates from the postgraduate programs, and (78%) reported the demand was “high.”

### Administration

**Fig. 5:**
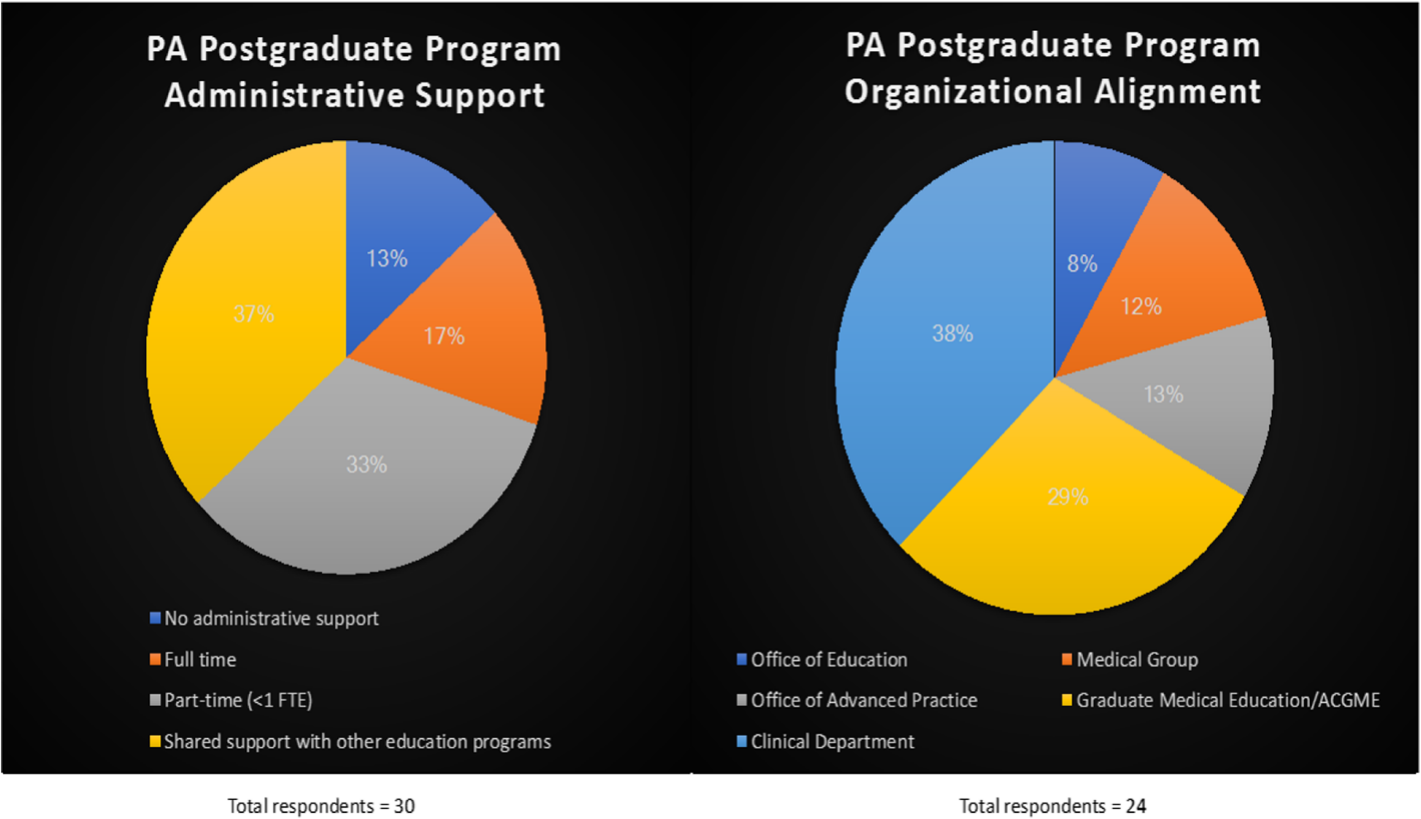
PA Postgraduate Program Administration

The majority of postgraduate programs (90%) reported that the chief administrator was a PA. One PA reported being a full-time administrator of multiple tracks, with the remainder varying from 4 h to 20 h a week of single-track programs (63%). Most (80%) of the PA administrators reported 5 years or more of clinical experience before assuming the program director role. In addition, a medical director was assigned to all PA postgraduate programs. The role of the medical director serves as the main medical supervisor of the PA fellow with involvement in educational assessment and program advocacy. The majority (76%) of physician medical directors are not allocated additional administrative time outside their clinical responsibilities. In most cases, physician supervision is a requirement of PA state licensure. A majority of programs have administrative support. Programs differed administratively by clinical department (38%), office of Graduate Medical Education (29%), office of ‘advanced practice’ (13%), medical group (13%), or other (7%). Figure 5**.**

### Organizational costs associated with the PA postgraduate programs

The majority of costs associated with the PA postgraduate education were derived from the medical group (42%) or the hospital system (46%) with the remainder from graduate medical education or a private donor (12%). Also, 90% postgraduate programs reported that they do no pay an honorarium or stipend to clinical faculty to supervise postgraduate APP trainees in the clinical setting. Lastly, administrative costs to keep the program operational were not studied.

### Billing for PA postgraduate trainee services

PAs enrolled in postgraduate education are NCCPA-certified, licensed in the state where the program resides, and eligible for billing of their services. Billing is done by the hospital, medical group, or academic medical center and most (79%) reported billing for services provided by the PA fellow. Additionally, four program directors said they intended to begin billing for services rendered by their PAs. It should be noted that not all PA postgraduate programs bill for first assist services. Billing for these services typically requires an attestation from the surgeon that no qualified medical resident is available to assist with eligible operative cases. Medicare reimbursement for a PA/NP assisting in surgery is 13.6% of the primary surgeon’s allowable fee and some surgeries are ‘restricted’ from reimbursement.

### PA postgraduate trainee compensation and benefits

All programs provide a stipend for the PA fellow. The amount varied between $50,000–80,000 (2019 dollars). Omitted were the wages for federal employees (e.g., military and VHA) which are government scheduled and at a higher rate than civilian stipends. Employment benefits (not reported) include paid time off (2–4 weeks), health insurance, CME, liability insurance, and clothing/uniforms.

### ARC-PA accreditation

Accreditation of PA postgraduate programs has been evolving in various forms since 2010 but the process was put in “abeyance” in 2014. One reason for suspension was that not all programs participated in accreditation, and none were bound by the accreditation process. The following survey question was about an accreditation process. “*If ARC-PA accreditation becomes available for your program will you pursue it?”* Two-thirds (67%) said they would *decline*. Another two-thirds (68%) said they had discussed ARC-PA accreditation funding with their institution. The main reason for declining accreditation was cost (48%), as well as lack of support (18%), and other (joint PA/NP program or cost of multi-track programs). A plurality (37%) would be interested in applying for joint accreditation (not yet available) if it included NPs.

### Including nurse practitioners in APPAP (selected comment)

*“There is an undeniable need for a JOINT accreditation process … as more programs are accepting NP and PA applicants.”*

### Value of postgraduate programs at sponsoring institutions

To probe the reasons why programs have developed and remain operational, a series of questions were asked. Almost all agreed that recruitment and retention of postgraduate trainees are part of meeting workforce demands (83%), and retention of career staff (55%). Most program directors (76%) believed their program improved decision making and autonomy of PAs/NPs in the clinical setting. Additionally, (93%) of respondents believed their postgraduate program fostered interprofessional collaboration. Lastly, (34%) of respondents felt their postgraduate training program improved compliance with physician resident regulated work hours.

## Discussion

This is the first study to describe the broad characteristics of postgraduate PA development in the United States. These findings shed light on the demographic, administrative, and educational aspects associated with PA postgraduate education. This information is of particular interest given the rising number of PA postgraduate programs and the desire for PA specialty training opportunities to support emerging and changing healthcare care needs. Additionally, the findings can be used to support PA postgraduate program development and expansion, continuous process improvement, and perhaps inform future policy decisions. The findings narrow the gap in the literature concerning post-graduate PA education and provides a springboard for further study on its perceived utility.

In 2020, an estimated 200 enrollees have graduated from 72 programs [13]. Because program records have not been centralized, it is difficult to determine the total number of new programs versus those that have closed or suspended during the COVID-19 pandemic. As a result, a summary of the number of graduates from PA postgraduate programs is approximated. The number of PA graduates from 265 programs in 2020 numbered approximately 10,000 and we estimate about 200 entered a postgraduate program in 2020 [6].

Why did PA postgraduate programs develop and why do they remain operational? From the program director’s viewpoint, having a PA postgraduate program in clinical medicine and surgery provides the new PA an opportunity to focus on a specialty and receive intensive training in a structured and abbreviated period. Value for the employing hospital or medical practice is from providers who can also supplement the reduction in physician residency slots and limitations on resident work hours [13, 14]. PAs and NPs provide readily available sources of personnel for various roles in the healthcare system.

Administratively, adding a PA into an existing postgraduate training program is not likely to impact the ‘span of management’ for an attending physician. Adding another trainee at the margin appears to require little more in the human resource office or in department commitment [6]. From the student’s viewpoint, additional education with supportive mentors provides more confidence that the graduate will be gainfully employed with a marketable skill set [15]. The economist views additional education as an opportunity cost [16]. In essence for additional (optional) clinical education the PA foregoes full employment (at 50% of usual wages) for 1 year. The exception being the military where the trainee is also a full-time government employee. From the educator’s point of view the salary of a postgraduate trained PA does not differ from on-the-job trained PAs and therefore, some may be reluctant to recommend a PA postgraduate program [16, 17].

As for the future, based on this census and survey project, half of the respondent programs have been operational for more than 10 years. Older programs include Montefiore Hospital (Surgery, Critical Care), Norwalk Hospital/Yale Medical University (Burn/Surgery), The Army/Air Force-Baylor doctoral program (Orthopedics, Emergency Medicine, General Surgery/Intensivist) and Arrowhead Orthopedic Surgery PA Fellowship program. Most of the programs (74%) provide experience in the inpatient and outpatient settings. A growing number of institutions, such as The Mayo Clinic, have multiple postgraduate programs for PAs and NPs located on three campuses suggesting their demand is growing in large medical centers [4, 18]. The VHA, a vertically integrated medical system within the Department of Veterans Affairs, has eight postgraduate programs for PAs and NPs ranging from primary care, geriatrics, psychiatry, to orthopedics [17]. Given the VHA has 160 medical centers and most are affiliated with an academic medical center, the growth of PAs and NP staffing is likely to continue [18].

### Limitations

All surveys have limitations and this one was no exception. The APPAP survey was lengthy (average 17 min), which may have contributed to response fatigue and a low rate of return (47%). Online surveys capture convenient samples that may or may not be representative ones with varying rates of survey responses that may lead to the potential for bias. In addition, several clinically focused educational programs were suspended, or activity restricted, during the COVID-19 pandemic. This may have affected the ability or interest of program personnel to complete the survey.

One shortcoming is the limited information about NP postgraduate residencies in this census. The first NP residency began in 2007 [19] and centralized information of postgraduate NPs is in development but not available at the time of this project.

Another limitation is that it was a single-mode survey delivered by email invitation. No incentives were offered even though they can improve response rates in certain situations. The strength of this survey is its uniqueness in providing a contemporary overview of the characteristics of American clinical postgraduate education.

## Conclusion

The movement to enhance the graduate PA with additional clinical education began in 1973. A half century later, PA postgraduate programs remain a viable option for licensed PAs seeking specialty training in a variety of medical and surgical disciplines. Those most represented are surgery, emergency medicine, critical care, and orthopaedics. Most postgraduate programs are one year in length and utilize the PA as house officer alongside categorical residents. The 34 participating PA program directors in this APPAP member survey have several elements in common as they align with General Medical Education approved postgraduate programs and the PA is added at the margin of infrastructure. The prediction is that PA and NP postgraduate programs will grow as the shortfall in medical and surgical specialties worsens. As a source of readily available and reliable medical and surgical workforce their addition appears to be a necessity. Areas of needed research include return on investment for the sponsoring institution, external funding (i.e.*,* grants) opportunities for postgraduate PA programs, assessing the career arc of PA/NP graduates, comparing postgraduate to on-the-job trainees, and assessing the opportunity cost of PA and NP postgraduate programs.

## Supplementary Information

**Additional file 1.**

## Data Availability

Available from the corresponding author and APPAP Survey committee on reasonable request
