# Supplementary material for: A National Survey of postgraduate physician assistant fellowship and residency programs
Source: BMC Med Educ. 2021 Apr 14;21:212. doi: 10.1186/s12909-021-02613-y (PMC8045993; doi:10.1186/s12909-021-02613-y)
Supplement: Supplementary file 1 — Additional file 1. [file 12909_2021_2613_MOESM1_ESM.pdf]

DEMOGRAPHICS

* 1. Program Name: *(optional)*

* 2. Location:(Dropdown)

3. Is your program:

- PA - Physician Assistant
- NP - Nurse Practitioner
- NP and PA

4. Type of program:

- Single Program
- Multi-track

5. The program is affiliated to a:

- PA School
- Nursing School
- Medical School
- Health Sciences

Other (please specify)

6. Hospital/Clinic affiliation:

- Academic Medical Center
- Multi-hospital system
- Community hospital
- VA
- Ambulatory clinic

Other (please specify)

7. Duration of Program:

- ◯ 9 months
- ◯ 12 months
- ◯ 18 months
- ◯ 2 years

Other (please specify)

8. Our program has been established for:

- ◯ Just underdevelopment, no graduates yet
- ◯ 1-3 years
- ◯ 4-6 Years
- ◯ 7-10 Years
- ◯ More than 10 years

9. Does your postgraduate program offer training in the:

- ◯ Inpatient setting only
- ◯ Outpatient setting only
- ◯ Both inpatient and outpatient settings

10. Select all specialty tracks that apply

- ☐ Inpatient/Hospitalist
- ☐ Surgical
- ☐ Emergency Medicine
- ☐ Orthopaedics
- ☐ Critical Care
- ☐ Psychiatry and Behavioral Health
- ☐ Hematology/Oncology
- ☐ Primary Care/Family Practice
- ☐ Trauma
- ☐ Pediatrics
- ☐ Cardiology/Heart failure
- ☐ Dermatology
- ☐ Other (please specify)

11. On completion of the postgraduate program, graduates receive:

- Certificate of completion
- Degree
- Diploma
- No formal award or certification

PA FELLOW/RESIDENT DEMOGRAPHICS

12. Are your APP postgraduate trainees referred to as residents or fellows?

- ○ Residents
- ○ Fellows

Other (please specify)

13. What % of fellows are employed by 2 months post-graduation?

0 100

14. How would you quantify the demand by employers for APP graduates from your fellowship/residency program?

- ○ There is a high demand by employers for our PA/NP graduates
- ○ There is a moderate demand by employers for our PA/NP graduates
- ○ There is a low demand by employers for our PA/NP graduates

15. Do your postgraduate APP trainees and/or faculty help clinically precept PA and/or NP students?

- ○ Yes
- ○ No

16. Do your APP trainees work alongside MD/DO residents in the same specialty?

- ○ Yes
- ○ No

17. Is your applicant pool: (select all that apply)

- ☐ Local
- ☐ Regional
- ☐ National

18. What percentage of applicants to your APP postgraduate programs are recent PA graduates (within 2 years of PA/NP graduation)?

0 100

19. Are your postgraduate trainees credentialed and privileged similar to other PA in the institution?

- Yes
- No

EDUCATION & CLINICAL TRAINING

20. Does your program offer the following education delivery methods for your APP trainees? *(Select all that apply)*

- ☐ Simulation
- ☐ Case studies
- ☐ Bedside clinical teaching
- ☐ In person didactic/lectures
- ☐ Online learning modules
- ☐ Grand rounds and/or conferences
- ☐ Anatomy lab
- ☐ Skills labs
- ☐ Journal clubs
- ☐ Core clinical rotations related to the specialty
- ☐ Research
- ☐ Networking with other fellows
- ☐ Mentored specialty projects

Other (please specify)

____

21. Beyond specialty education, the program incorporates *(Select all that apply)*

- [ ] Professionalism/ Emotional Intelligence
- [ ] Specialty curriculum
- [ ] Procedure training
- [ ] Core education curriculum – broader topics than program specialty
- [ ] Research curriculum
- [ ] Quality improvement curriculum
- [ ] Wellness & Resiliency
- [ ] Career/Professional advancement education
- [ ] Ethics
- [ ] Information Technology
- [ ] Team-based Care
- [ ] Interpersonal communication
- [ ] Scholarly writing & publication
- [ ] Team-based care
- [ ] PA/NP practice, utilization, and regulations

22. Are your APP trainees given access to medical educational resources (ie: library access, lecture material, etc) as physician residents at your institution?

- ○ Yes
- ○ No

23. Do you use electronic systems for: *(Select all that apply)*

- [ ] Delivering electronic education
- [ ] Evaluations
- [ ] Electronic medical records

Other (please specify)

24. Evaluations and assessments of the fellows are done in the following methods: *(Select all that apply)*

- [ ] 1:1 clinical observation
- [ ] Competency simulation
- [ ] Achieving clinical milestones
- [ ] Completion of program requirements
- [ ] Regular reviews
- [ ] Achieving minimal score on a multiple choice test
- [ ] Minimum proficient procedures
- [ ] Preceptor evaluations
- [ ] Self-assessment
- [ ] Other (please specify)

ACCREDITATION

25. Are you planning on pursuing ARC-PA accreditation for your postgraduate fellowship or residency program when the process becomes available later this year? *(arc-pa.org)*

- ◯ Yes
- ◯ No

26. Have you had conversations in your institution's financial structure regarding funding accreditation?

- ◯ Yes
- ◯ No

27. If Yes, what are your reasons for pursuing accreditation?

- ◯ Program quality standards
- ◯ Requirement of institution
- ◯ Competition for applications

Other (please specify)

[ ]

28. If No, what is your reason for not pursuing accreditation?

- ◯ Lack of support/interest
- ◯ Cost of one program
- ◯ Cost of multi-track programs
- ◯ It is a joint NP/PA program

29. If a joint accreditation for NP and PA was available, would your program would you apply for joint accreditation?

- ◯ Yes, as a single track program
- ◯ Yes, as a multi-track program
- ◯ No, we are only a PA program
- ◯ No, not interested in accreditation

FELLOWSHIP/RESIDENCY LEADERSHIP DEMOGRAPHICS

30. Is there a person assigned exclusively for the provision of administrative and/or secretarial support for the program and is this person full time or part-time?

- ◯ Full time (1.0 FTE)
- ◯ Part-time (<1.0 FTE)
- ◯ There is shared administration with other education or programs
- ◯ There is no person assigned to provide administrative and/or secretarial support for the program

31. Is your program connected to the following organizational offices?

- ◯ Office of Advanced Practice
- ◯ Clinical Department
- ◯ Medical group
- ◯ Office of Education
- ◯ Graduate Medical Education/ACGME

Other (please specify)

[ ]

32. Does your program have a central PA/NP fellowship leadership that oversees, provides standards/structure for all postgraduate programs?

- ◯ Yes
- ◯ No
- ◯ Does not apply because it is a single track program.

33. As a PA Program Director, what % FTE are you given administrative time to manage the program?

- ◯ FTE 0.1 (4 hours a week)
- ◯ FTE 0.2 (8 hours a week)
- ◯ FTE 0.4 (16 hours a week)
- ◯ FTE 0.5 (20 hours a week)
- ◯ FTE 0.6 (24 hours a week)
- ◯ FTE 0.8 (32 hours a week)
- ◯ FTE – Full time
- ◯ None

34. How much clinical experience did you have before assuming the role of APP fellowship director?

- 1 year or less
- 2-4 years
- 5-7 years
- 8-10 years
- 11-15 years
- > 15 years

35. What is the role of your MD program director?

- MD sponsorship
- Education
- Clinical skill assessment
- Candidate selection
- Other

36. Is your MD program director given FTE allocation?

- FTE (4 hours a week)
- FTE (8 hours a week)
- FTE (16 hours a week)
- FTE (20 hours a week)
- FTE (24 hours a week)
- 0.8 FTE (32 hours a week)
- FTE – Full time
- None

PROGRAM FINANCIALS

37. Who sponsors the majority of the costs associated with the training of postgraduate APPs at your institution?

- ◯ Medical Group
- ◯ GME
- ◯ Hospital System
- ◯ Private Donors

Other (please specify)

38. Does your program receive external funding from grants?

- ◯ HRSA
- ◯ GME

Other (please specify)

39. Do you bill for the services provided by your APP trainees?

- ◯ Yes
- ◯ No

40. If you do not bill for services provided by your APP trainees currently, are you planning to bill in the future? *(Note: If you are a program with multiple clinical tracks and bill for some programs but not others, please select YES to this question).*

- ◯ Yes
- ◯ No

41. If you were eligible to receive external funding (grants, etc.), what would you use the money for? (Check all that apply)

- ☐ APP training stipend
- ☐ Administrative support
- ☐ Fringe benefits
- ☐ Hiring more clinical faculty
- ☐ Hiring more didactic faculty
- ☐ Recruiting and marketing
- ☐ Purchasing required learning materials
- ☐ Creating additional APP training fellowships
- ☐ Research activities that lead to publications
- ☐ Procuring additional clinical sites for expanded training opportunities
- ☐ Accreditation costs

42. What benefits and compensation are offered to the postgraduate trainees?

- ☐ Stipend
- ☐ Health benefits
- ☐ CME funds
- ☐ Free educational CME
- ☐ Education resources
- ☐ Loan deferment
- ☐ Vacation time accrual
- ☐ Time away – sick time, holiday, conference, etc.
- ☐ Tuition reimbursement
- ☐ Retention bonus

Other (please specify)

[ ]

43. What is the stipend provided to your postgraduate training annually

- ○ Less than $50,000
- ○ $50,000 – 59,999
- ○ $60,000 – 69,999
- ○ $70,000 – 79,999
- ○ $80,000 +

44. Do you pay an honorarium or stipend to clinical faculty to supervise postgraduate APP trainees in the clinical setting?

- ◯ Yes, we pay clinical faculty to supervise our postgraduate APP trainees in the clinical setting.
- ◯ No, we do not pay clinical faculty to supervise our postgraduate APP trainees in the clinical setting.

45. Do you pay an honorarium or stipend to faculty to provide lectures to postgraduate APP trainees at your institution?

- ◯ Yes, we pay clinical faculty to provide lectures to our postgraduate APP trainees.
- ◯ No, we do not pay clinical faculty to provide lectures to our postgraduate APP trainees.

46. What critical needs/ return on investment does postgraduate training demonstrate at your institution? (Check all that apply)

- ☐ Recruitment and retention of postgraduate trainees
- ☐ Professional development/retention of current APPs
- ☐ Specialty training of APPs
- ☐ Primary care training of APPs
- ☐ Improving independent decision making and autonomy of APPs in the clinical setting
- ☐ Increasing access to medical services
- ☐ Decreasing patient wait times
- ☐ Meeting workforce demands
- ☐ Clinical precepting of health professional students rotating in the medical specialty
- ☐ Expanding additional service lines
- ☐ Improving compliance with physician resident ACGME regulated work hours

Other (please specify)

____

47. Does having APP postgraduate training programs at your institution foster interprofessional collaboration?

- ◯ Yes
- ◯ No

APPAP MEMBERSHIP VALUE

48. How long has the fellowship program been part of APPAP?

- ○ 0 to < 1 years
- ○ 1 to < 3 years
- ○ 3- 5 years
- ○ 5 – 10 years
- ○ More than 10 years

49. What was the initial reason for joining APPAP? *(Select all that apply)*

- ☐ Networking with other programs
- ☐ APPAP/APGAP conference
- ☐ Bi-annual meetings
- ☐ Online program resources
- ☐ Program listing on the website
- ☐ Research opportunities
- ☐ Updated postgraduate research resources
- ☐ Job listings on the website
- ☐ Website
- ☐ Awards
- ☐ Accreditation updates
- ☐ Resources for postgraduate program graduates
- ☐ National leadership opportunities

Other (please specify)

[ ]

50. What are the top reasons you continue to maintain the membership? (Limit 3)

- [ ] APPAP/APGAP conference
- [ ] Bi-annual business meetings
- [ ] Online program resources
- [ ] Program listing on the website
- [ ] Research opportunities
- [ ] Updated postgraduate research resources
- [ ] Job listings on the website
- [ ] Website
- [ ] Awards
- [ ] Accreditation updates
- [ ] Resources for postgraduate program graduates
- [ ] National leadership opportunities

Other (please specify)

51. How satisfied is your program with the offerings from APPAP?

	Very Satisfied	Satisfied	Neutral	Dissatisfied	Very Dissatisfied
Networking with other programs	○	○	○	○	○
APPAP/APGAP conference	○	○	○	○	○
Biannual meetings	○	○	○	○	○
Online resources & research	○	○	○	○	○
Program listing on the website	○	○	○	○	○
Research opportunities	○	○	○	○	○
Job listings on the website	○	○	○	○	○
Website	○	○	○	○	○
Awards	○	○	○	○	○
Accreditation updates	○	○	○	○	○
Resources for PA program graduates	○	○	○	○	○
National leadership opportunities	○	○	○	○	○

52. Is your program planning on renewing the APPAP membership in 2021?

- Yes
- No

If no, what are the reasons for not renewing?

53. Are you a member of APGAP?

- Yes
- No

If yes, what benefits and resources do APGAP provide that are not available through APPAP?

54. If yes, how satisfied are you with the website format?

Very Satisfied	Satisfied	Dissatisfied	Very Dissatisfied
○	○	○	○

55. Comments or feedback on the website?

56. Did you attend the joint APPAP/APGAP event in January, ADVANCED PRACTICE PROVIDER FELLOWSHIP WORKSHOP: LAUNCHING & REFINING YOUR PROGRAMS?

- Yes
- No

Reason for attending or not attending.

57. What would you like APPAP to offer that is currently not offered?

58. Do you have recommendations to increase membership or value of APPAP?

59. Additional Comments:
